# Supplementary material for: Kinetics and detectability of the bridgmanite to post-perovskite transformation in the Earth's D″ layer
Source: Nat Commun. 2019 Dec 12;10:5680. doi: 10.1038/s41467-019-13482-x (PMC6908603; doi:10.1038/s41467-019-13482-x)
Supplement: Supplementary file 2 — Supplementary Information [file 41467_2019_13482_MOESM2_ESM.pdf]

Supplementary Information

Kinetics and detectability of the bridgmanite to post-perovskite transformation in the Earth's layer

Langrand et al.

Conditions	Time (s)	Fraction of pPv (wt%)	ξ
126 GPa, 1950 K	0	21	0.00
	114	26	0.58
	233	26	0.69
	353	28	0.90
	472	29	0.96
	599	29	1.00
126 GPa, 2100 K	0	0	0.00
	20	0	0.00
	79	14	0.29
	123	31	0.62
	149	31	0.62
	167	35	0.71
	208	37	0.75
	257	44	0.89
	291	48	0.96
	351	49	0.98
	412	49	0.99
	480	50	1.00
126 GPa, 2400 K	0	33	0.00
	68	34	0.06
	120	42	1.03
	155	43	1.19
	173	42	1.02
	190	41	0.89
	205	40	0.84
	218	42	1.08
	233	42	1.07
	246	40	0.82
	264	42	1.03

Supplementary Table I: Weight proportion of pPv and extend of transformation vs. time for all experiments performed at 126 GPa.

Conditions	Time (s)	Fraction of pPv (wt%)	ξ
129.5 GPa, 1600 K	0	7	0.00
	215	16	0.17
	470	38	0.58
	535	44	0.70
	581	47	0.75
	739	51	0.83
	1020	55	0.91
	1159	60	1.00
	1288	60	1.00
129.5 GPa, 1700 K	0	12	0.04
	52	16	0.12
	83	24	0.28
	113	28	0.36
	124	32	0.43
	144	35	0.49
	174	42	0.63
	200	44	0.67
	231	48	0.74
	321	52	0.82
	392	57	0.93
	431	61	1.00
129.5 GPa, 1850 K	0	48	0.00
	29	65	0.43
	37	69	0.54
	52	76	0.72
	77	81	0.86
	139	87	1.00

Supplementary Table II: Weight proportion of pPv and extend of transformation vs. time for all experiments performed at 129.5 GPa.

Phase boundary			Nucleation and growth			Shear transformation		
α (MPa.K^{-1})	P_e (GPa)	T_e (K)	Q_0 (kJ.mol^{-1})	$\ln k_2$ (s.K)	V^* (cm^3.mol^{-1})	Q_0 (kJ.mol^{-1})	$\ln k_2$ (s)	V^* (cm^3.mol^{-1})
8.5	123	3300	474(141)	-17.3(6.1)	15.3(5.8)	477(148)	-17.7(6.4)	16.1(6.1)
6.5	128	3300	369(102)	-15.6(5.1)	13.6(5.6)	382(112)	-15.7(5.6)	16.1(6.1)
8.5	*128*	*3300*	*426(122)*	*-17.5(5.8)*	*14.4(5.6)*	*437(132)*	*-17.7(6.3)*	*16.1(6.1)*
10.5	128	3300	484(142)	-19.4(6.6)	15.0(5.7)	490(152)	-19.6(7.1)	16.1(6.1)
8.5	133	3300	428(112)	-20.6(6.0)	14.7(6.0)	406(109)	-18.3(5.9)	16.5(5.8)

Supplementary Table III: Transformation kinetics models with different locations of the Pv/Pv+pPv phase boundary defined by its Clapeyron slope (α) a reference point [P_e,T_e] belonging to the boundary. Numbers in parenthesis are 1σ uncertainties on the fitted values. Numbers in italics indicate the reference phase boundary used for the main figures in the manuscript. The effect of varying (P_e,T_e) is shown in Supplementary Figs. 4 and 5. Errors in the main manuscript account for a ±5 GPa error in absolute pressure and ±2 MPa.K^{-1} for the Clapeyron slope

.

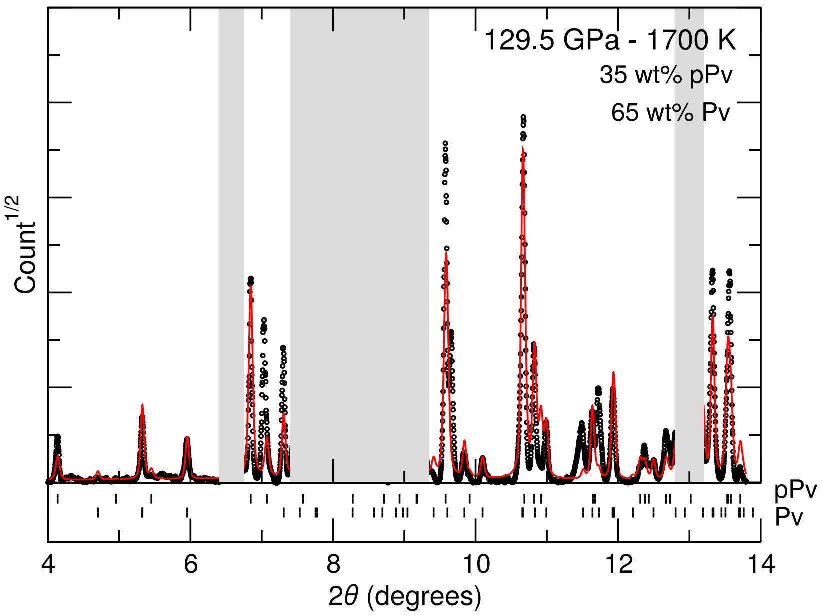

Supplementary Figure 1: Sample Rietveld refinement for a mixed Pv-pPv sample at 129.5 GPa and 1700 K with 35 and 65 wt% of pPv and Pv, respectively. Black dots are experimental data, red line is recalculated based on the Rietveld refinement. Grey shadings indicate regions that were ignored in the refinements because of the presence of intense diffraction peaks from the Ar pressure medium. Ticks at the bottom of the plot indicate the location of the diffraction peaks of Pv and pPv.

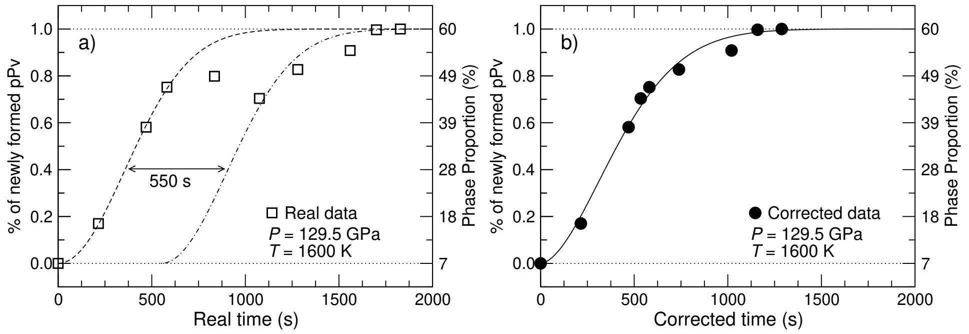

Supplementary Figure 2: Correction for diamond anvil cell movements at P = 129.5 GPa and T = 1600 K. *a)* Extend of transformation vs. time measured in the experiment. The sample alignment with lasers and x-rays is lost due to a shift of the diamond anvil cell after 600 s and corrected after 1000 s by moving the sample back in front of the x-ray and laser beams. During this interval, the transformation is stopped in the sample's region of interest. The data before 600 s is fitted to the Avrami model to estimate a first value of n and τ (dashed line). A delay time is then fitted for the data collected after 1000 s using the same n and τ values. Both datasets are then fitted, accounting for the delay time, to extract new values for n and τ. The procedure is repeated until convergence for the values of the delay time, n and τ. The final value for delay time is 550 s. *b)* Corrected dataset with the corresponding adjustment of an Avrami model. The first estimate of τ is 498 s. The final result is $\tau = 502$ s.

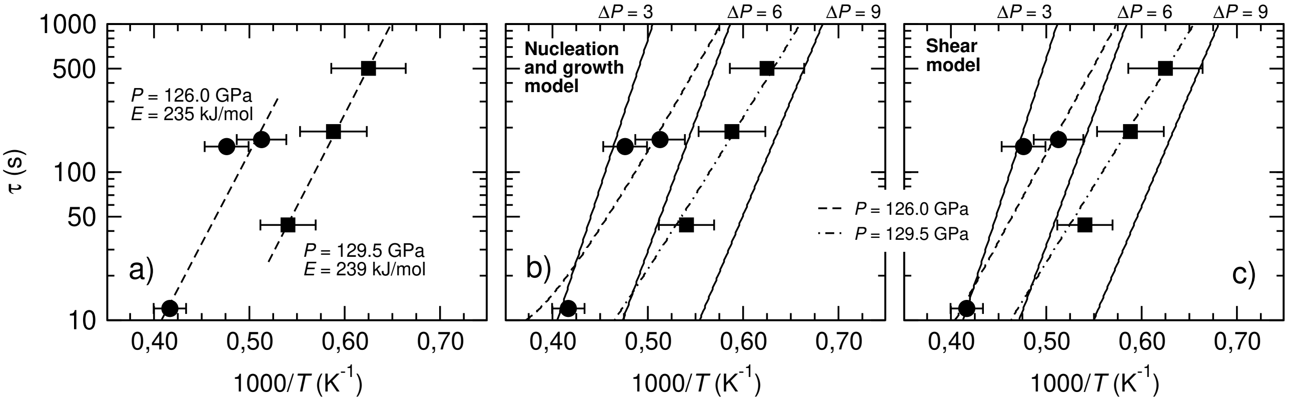

Supplementary Figure 3: a) Transformation characteristic time vs. inverse temperature and extraction of apparent activation energies. Circles and squares are experimental data at 126.0 GPa and 129.5 GPa, respectively. Dashed lines are fit results of an Arrhenius equation for each pressure. The Arrhenius equation accounts for the effect of temperature but does not model the effect of the over-pressure from the phase boundary. b,c) Results of nucleation and growth and shear model with $P_e = 128$ GPa, $T_e = 3300$ GPa and $\alpha = 8.5$ MPa.K^{-1}. Solid lines are model results at constant overpressure (i.e. distance from the phase boundary, with $\Delta P = 3$, 6, and 9 GPa). Dashed lines are model results at constant pressure (as shown in Fig. a).

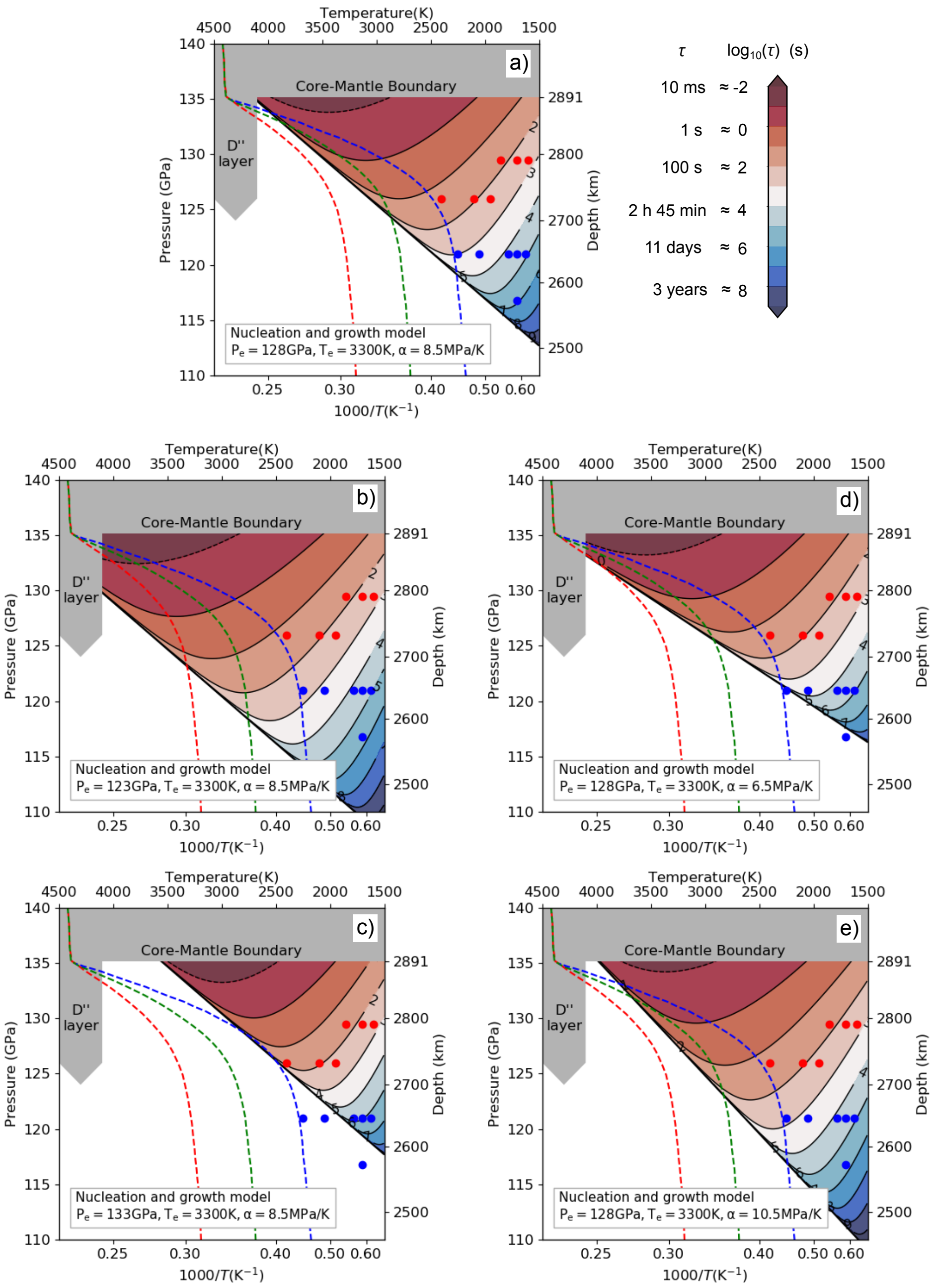

Supplementary Figure 4: Effect of the location of the Pv/Pv+pPv phase boundary on the extrapolations of the nucleation and growth model. Thick solid line: Pv/Pv+pPv equilibrium boundary. Color scale: characteristic transformation times. Red, green, and blue dashed line: hot, warm and cold geotherms of Ref. 5. Red dots: P/T points with transformation between Pv and pPv. Blue dots: P/T points with no transformation between Pv and pPv. Fig. A is that of the main paper, with a reference point on the Pv/Pv+pPv equilibrium boundary at $T_e = 3000$ K and $P_e = 128$ GPa and a Clapeyron slope of 8.5 MPa.K^{-1}. Figs. B and C show the effect of a ± 5 GPa change in P_e. Figs. D abd E show the effect of a ± 2 MPa.K^{-1} change for the Clapeyron slope. Detailed fit parameters for all figures are listed in Supplementary Table III. Pressure (P_e), temperature (T_e) and Clapeyron slope (α) defining the Pv/Pv+pPv equilibrium boundary are indicated in the inset of each figure.

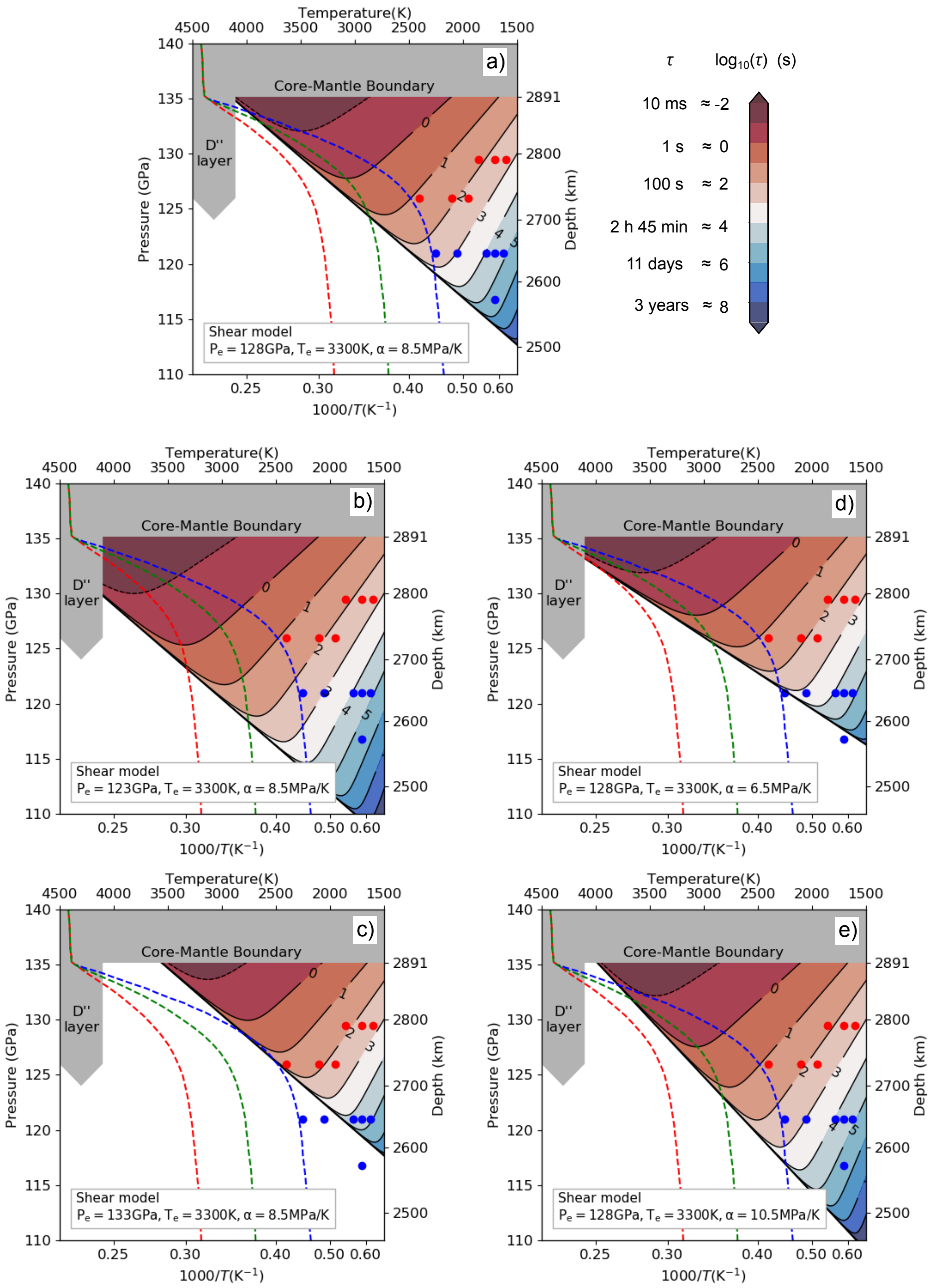

Supplementary Figure 5: Same as Suppl. Fig. 4 but for the shear transformation model. Pressure (P_e), temperature (T_e) and Clapeyron slope (α) defining the Pv/Pv+pPv equilibrium boundary are indicated in the inset of each figure.

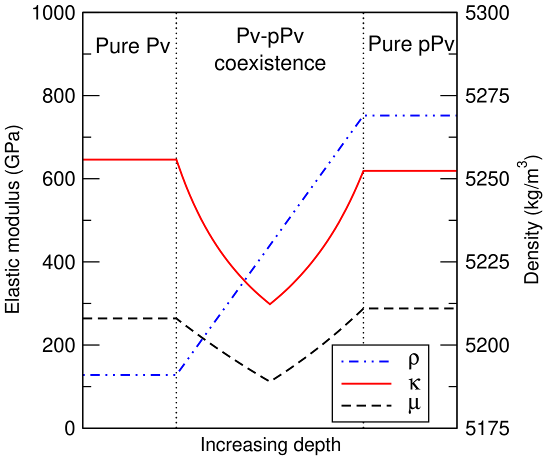

Supplementary Figure 6: Effective bulk modulus κ, shear modulus μ and density ρ as a function of depth. Single phase data were computed at 128 GPa and 2800 K[37]. The effective elastic moduli are reduced in the phase transformation depth range due to the kinetics of the phase transformation[23,24].
